# Modelling climatic and temporal dynamics of dengue transmission in Bangladesh using deep learning models

**DOI:** 10.1371/journal.pgph.0006405

**Published:** 2026-07-13

**Authors:** Mahadee Al Mobin, Arju Manara Begum

**Affiliations:** Bangladesh Institute of Governance and Management, Dhaka, Bangladesh; University of Hong Kong, HONG KONG

## Abstract

Dengue fever is a significant public health issue in tropical and subtropical areas, and predicting its spread is challenging due to the complex interactions between climate factors, mosquito behavior, and case reporting. This study develops a high-resolution forecasting framework that integrates daily dengue case data with meteorological drivers (including mean, maximum, and minimum temperature; precipitation; specific and relative humidity; surface pressure; mean, minimum, and maximum wind speed; wind speed range; wind direction; and sunshine duration) to improve predictions in Bangladesh. Aggregated case counts were disaggregated using a Stochastic Bayesian Downscaling (SBD) algorithm, followed by systematic feature engineering. A wide range of deep learning models, including Artificial Neural Networks (ANN), recurrent networks (LSTM, GRU, BiLSTM, BiGRU), attention-based models, and hybrid convolution neural network (CNN) based ensembles, were optimized through Bayesian hyperparameter tuning and evaluated under a unified process. Results demonstrated that a simple ANN achieved the highest performance, with an accuracy of 97.05%, RMSE of 145.02, and MAPE of 0.51%, surpassing more complex recurrent and attention-based models. Feature importance analysis revealed that weather variables accounted for 76.2% of predictive accuracy, with lagged climate features contributing 59.4%. Short-term lags of three to seven days proved especially influential, underscoring the importance of near-real-time weather monitoring. The strongest predictors included surface pressure with a 3-day lag, precipitation, and maximum wind speed with 7-day lags, while measures of variability such as rolling standard deviations also contributed. The study highlights three key contributions: the benefit of combining downscaling, lagged climate features, and systematic feature selection; evidence that ANN models can outperform more complex architectures in dengue forecasting; and the identification of short-lag weather factors that can support early warning systems. The framework provides an efficient, reproducible, and scalable approach that can strengthen dengue preparedness in public health systems with limited resources.

## Introduction

Dengue is a vector borne viral disease that has now been transmitted to more than 120 countries worldwide [1]. Each year, nearly 400 million cases are reported, mainly in urban and peri-urban areas of tropical and subtropical regions [1]. This concerning trend highlights the urgent need for better monitoring, forecasting, and control methods.

The patterns of dengue outbreaks resemble those of other diseases spread by vectors. These patterns result from complex interactions among hosts, vectors, and viruses, which are also influenced by environmental and climatic conditions [2,3]. Changes in climate are often considered a major factor in dengue cases, but their effects differ by location. For instance, studies in China and Vietnam have found a strong positive connection between rainfall and dengue cases, with delays of up to three months [4–9]. In contrast, research in other areas has found either weak or negative associations [10–12].

Temperature is also important. Minimum temperature often acts as a strong predictor of dengue incidence, typically with lags of one to two months [4,8,10,13,14]. Average monthly or weekly temperatures can show positive correlations with dengue cases over shorter lags of 0 to 2.5 months [5–7], though some findings conflict with this [15]. Studies on relative humidity show mixed results. Some find positive links [4,7,8,10], while others suggest negative or weak connections [5]. Interestingly, when examining delays of one to three months, relative humidity appears to have a more consistent relationship with dengue cases [4,8]. Factors such as sunshine duration, wind speed, evaporation [5], and El Niño events [10] have also produced mixed results. These inconsistencies highlight the complex interactions between climate and dengue.

Recently, deep learning (DL) methods have proven effective in predicting dengue because they can capture complex relationships in climate and health systems. For example, Xinxing et al. [16] created a hybrid model that combines recurrent neural networks (RNN) and CNN to forecast weekly dengue cases in Singapore. They achieved an accuracy of 87.72% for one-week predictions. However, this work focused only on short-term forecasts, lasting up to four weeks. Similarly, Nguyen et al. [17] used CNNs, Transformers, and LSTMs with dengue data from Vietnam. They found that attention-based LSTM (LSTM-ATT) models performed the best. However, their analysis was based on just 36 months of data and did not reveal actual incidence patterns.

Other important work comes from Xu et al [18]. They created an LSTM model covering 20 Chinese cities and achieved significant improvements over traditional models. Their method struggled in low-incidence areas and did not take into account cumulative or lagged effects of climate. Guo et al. [19] compared various models using dengue cases, search queries, and weather data in China, identifying SVR as the best performer. While it was effective, SVR is computationally demanding, making it hard to scale. Internet search data can also introduce biases, especially in rural areas.

Beyond DL, statistical and hybrid methods have been researched. Shi et al. [20] used LASSO regression in Singapore and found it was more accurate than SARIMA for many forecasting periods. Machuca et al. [21] improved LSTM predictions by applying clustering techniques to different time series. Jayaraj et al. [11] found that SARIMA was particularly effective for short-term forecasts in Malaysia. Patra et al. [22] used a CNN-BiLSTM hybrid on a decade of data in Laos, achieving high predictive performance. However, they ignored external weather variables, which raised concerns about overfitting.

Bangladesh has faced ongoing dengue problems since 2000. Yearly case numbers have fluctuated, with more severe outbreaks in recent years [23]. The crisis peaked in 2023, when the country reported its worst epidemic with 321,179 confirmed cases and 1,705 deaths. This was the highest burden ever recorded [24]. The outbreak has drawn a lot of attention from researchers and policymakers. It has led to several forecasting efforts to understand and manage dengue in Bangladesh.

Hossain et al. [14] were among the first to use generalized linear models (GLMs) to predict annual dengue cases by including seasonal climate variables such as minimum temperature, rainfall, and sunshine. Their findings showed a complex relationship. Minimum temperature had a positive impact on dengue cases in late winter but a negative one in early summer. This points to the need for models that can capture changing climate effects across the seasons. Although the study provides important insights, it is limited by the unavailability of 2019 meteorological data, which prevented the inclusion of that year’s outbreak. In addition, the use of only 19 annual data points (2010–2018) increases the risk of overfitting, especially given the relatively large number of predictors.

Dey et al. [25] expanded the methods by using Support Vector Regression (SVR) and Multiple Linear Regression (MLR) across 11 districts from August 2021 to May 2022. SVR consistently outperformed MLR, reaching 75% accuracy with an average error of 4.95. However, SVR’s scalability is limited because it requires a lot of computing power. The study also work on limited time frame of data largely ignoring the largest outbreak of 2019 till date, necessarily failing to capture dynamics of dengue outbreak and did not incorporated sophisticated methods to capture the intricate dynamics, which leaves room for improvement.

Mobin et al. [26] addressed data scarcity in forecasting by introducing the Mahadee Kamrujjaman Downscaling (MKD) algorithm, which is also referred to as the Stochastic Bayesian Downscaling method. They broke down aggregated data into smaller time frames, which led to better accuracy. SARIMA models trained on this improved data achieved up to 72.76% more accuracy than those using the original data. While this work was innovative, it only looked at case counts and did not include important weather variables.

Afterward, Mobin [27] developed a multivariate forecasting framework that included 13 weather variables along with dengue incidence data. By combining the SBD method with machine learning techniques like Random Forest, the study reached 95.85% predictive accuracy. They also used Granger causality and detailed feature engineering to clarify the time relationships among predictors. This represented a notable methodological advance. However, the focus was more on how data downscaling affected predictive performance rather than how climate factors influenced dengue spread. Additionally, the lack of deep learning models is a major limitation. This is especially true given the large-scale daily data available from January 2010 to December 2023.

Together, these studies have created a solid base for modeling dengue outbreak in Bangladesh. Yet, important gaps persist. Many studies depend on aggregated monthly data, which hides high-frequency dynamics vital for timely responses. Others overlook essential meteorological factors or fail to use strong feature selection techniques. Although downscaling methods show promise, their combination with deep learning models that can capture complex dynamics remains mostly largely unexplored. Filling these gaps is essential for developing a early warning system that is both precise and helpful for public health authorities in exploring the intricate impact of climatic drivers on the dengue outbreak especially in a low resources settings like Bangladesh.

Our study addresses these gaps by merging deep learning techniques with detailed climate and epidemiological data for Bangladesh. Specifically, we aim to contribute to the existing literature in several ways:

1. The paper uses the SBD method to convert aggregated counts into daily series. It shows that this method provides better feature engineering and model training than using only aggregated monthly data. This solves a common problem with data resolution in forecasting vector-borne diseases, especially in deep neural network (DNN) settings.
2. The authors implement and compare several DNN methods, including ANN, LSTM, GRU, BiLSTM, BiGRU, attention-augmented RNNs (LSTM-ATT, GRU-ATT), and CNN-RNN hybrid ensembles. They follow the same process for preprocessing, tuning, and evaluation. This thorough side-by-side comparison highlights the strengths of each model in relation to dengue time series.
3. The ANN achieved the highest reported test performance, with an accuracy of about 97.05%, RMSE of 145.02, and MAPE of 0.51%. This challenges the common belief that recurrent or attention models are always better for epidemiological sequences. It calls for a reconsideration of model choices with similar datasets.
4. The authors find that weather variables account for about 76.2% of model accuracy, while lagged weather predictors contribute around 59.4%. Short-term lags of 3–7 days show a strong influence, giving a clear statement about the timing of dengue transmission in the area. This both supports and sharpens existing expectations about mosquito life cycles and incubation periods.
5. The study shows that measures of variability, such as standard deviation, and very short rolling windows of 2 days perform better than intermediate windows and long seasonal windows. This finding is surprising but helpful for improving feature selection in outbreak forecasting.
6. The paper turns findings about feature importance into practical ideas for surveillance. It suggests monitoring surface pressure and short-term changes in rainfall and wind as early signs for vector control. This links modeling studies to decision-making support.

The rest of the manuscript is structures as follows: Materials and Method elaborates on the details of data, technical specification of the computational system, and methodological workflow of the study. Results discusses the performance of the DNN model, and findings from the feature selection analysis. Discussion presents the findings of the study through the lenses of epidemiology, policy, local context and global context. Conclusion presents the further avenues of research with the concluding remarks on the study.

## Materials and methods

### Data

The dataset of daily dengue case counts in Bangladesh, covering from 1st January 2010–31st December 2024, was sourced from the Directorate General of Health Services (DGHS), Bangladesh [28]. The Health Emergency Operation Center and Control Room of DGHS regularly gathers and shares daily data on dengue cases and related deaths across all administrative divisions of the country.

Meteorological factors, such as mean temperature, maximum and minimum temperature, specific humidity, relative humidity, precipitation, surface pressure, wind speed, maximum and minimum wind speed, wind speed range, and wind direction, were collected from the NASA [29]. Daily sunshine duration data were obtained from the Bangladesh Agricultural Research Council (BARC) [30]. A detailed description of these variables can be found in Table 1.

**Table 1:** Description of the temporal meteorological features of the dataset used for the study.

Sl.	Meteorological Variables	Short hand	Unit	Description
1	sunshine hours	sunshine (hour)	Hours	The average number of hours of sunshine recorded over a certain day.
2	Average daily temperature	temp	^*o*^ *C*	The average of the temperature of a day.
3	Maximum temperature	max_temp	^*o*^ *C*	The highest temperature recorded over a day.
4	Minimum temperature	min_temp	^*o*^ *C*	The lowest temperature recorded over a day.
5	Daily rainfall	precipitation	*mm*/*day*	Daily recorded precipitation.
6	Specific humidity	specific_humidity	*g*/*kg*	The average amount of water vapor in the air, measured over a day.
7	Relative humidity	relative_humidity	%	The relative humidity measured over a day.
8	Surface pressure	surface_pressure	*kPa*	The average atmospheric pressure at the surface level measured over a day.
9	Monthly average wind speed	wind_speed	*m*/*s*	The average wind speed measured over a month.
10	Minimum wind speed	min_wind_speed	*m*/*s*	The lowest wind speed recorded on average over a day.
11	Maximum wind speed	max_wind_speed	*m*/*s*	The highest wind speed recorded on average over a day.
12	Range of wind speed	range_wind_speed	*m*/*s*	The range of wind speeds measured over a day.
13	Direction of wind speed	wind_direction	*Degrees*	The direction of the wind measured over a day.

The table represents the description of data collected from NASA and BARC, with their unit and shorthand used in the dataset.

#### Technical specification.

All simulations presented in this study were conducted using Python (version 3.10.16) in conjunction with TensorFlow (version 2.10.1). The hardware specifications of the computational resources employed are detailed in Table 2.

**Table 2:** System specification for the experiment.

Component	Specification
RAM	64 GB
CPU	Intel i9-1400KF; 24 cores; 32 threads; 2.4 GHz
Cache	36 MB
Graphics	ZOTAC GAMING GeForce RTX 5080 SOLID; 16GB DDR7 Memory
Disk Space	1 TB

The table depicts the hardware specification of the computational setup used for the experimentation of the study.

### Methodology

**Fig 1:**
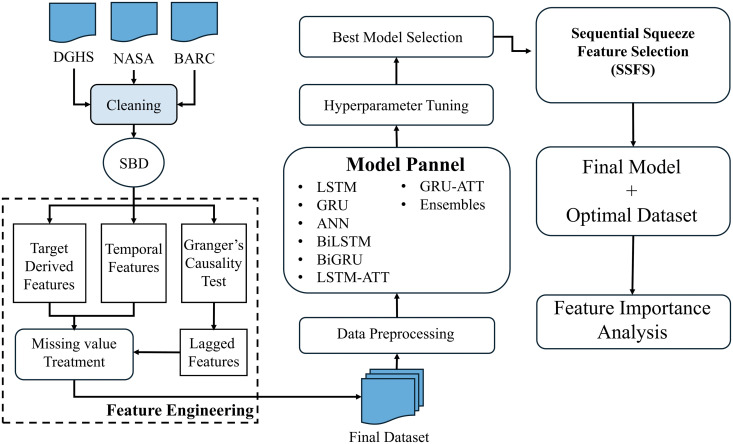
The methodological work flow of the study. The figure depicts the methodological workflow of the study. We initially collect the data from NASA, DGHS, and BARC. Of the collected data, we clean off any additional data which are not conducive to our cause. Based on the clean data set we conduct necessary feature engineering and remove any missing value created in the process, which leaves us with the final dataset. At this stage, we conduct data preprocessing (i.e., scaling the data. doing train-test split etc.) necessary to make the dataset ready for DL models followed by the selection of the best model from the panel based on validation score over the some predefined hyper parameter space for the final dataset.

In response to the rising number and intensity of dengue virus (DENV) outbreaks in Bangladesh, this investigation aims to create a strong forecasting framework, shown in Fig 1, that addresses this urgent public health issue. By using deep learning models designed for time series prediction, the proposed system seeks to produce accurate forecasts of upcoming outbreaks, focusing on thirteen key weather factors. A detailed description of these variables, along with their definitions, is provided in the “Data” subsection. This section outlines the steps taken to preprocess the dataset, starting with the initial cleaning phase and continuing through the following analysis stages.

#### Data cleaning.

The datasets obtained from the above sources were mostly clean, except for sunshine hours, which had a small percentage of missing values, around 2%. We filled in these gaps using the average value for the corresponding day, based on data from the previous three years.

**Fig 2:**
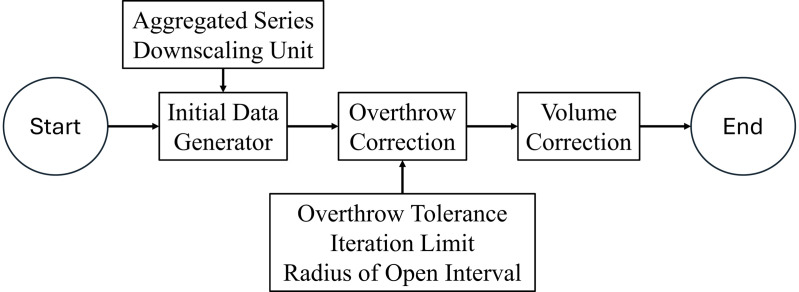
Workflow of the SBD algorithm. The figure depicts the methodological workflow of the stochastic Bayesian algorithm. The aggregated time along with the downscaled unit is placed in the initial generator which produces an initial guess for the downscaled data. Based on some predefined overthrow tolerance, iteration limit, and radius of open interval, SBD performs overthrow correction which in essence smooths out sharp spikes in the initial guess followed by a volume correction phase that ensures that the downscaled data agrees with the aggregated data in terms of sum of the data over the aggregated time unit.

To downscale the epidemiological records from 2010-2019 over time from monthly to daily scale, we used the Stochastic Bayesian Downscaling (SBD) algorithm [31]. This is a three-phase framework designed to create daily time series from aggregated data as illustrated in Fig 2. The statistical properties of SBD including its distributional fidelity relative to alternative disaggregation methods and its sensitivity to the overthrow tolerance parameter have been characterized in detail in the originating methodological work [31]. The forecasting implications of the downscaling step, including its effect on predictive accuracy relative to non-downscaled data, are evaluated in [27], where SBD-trained models showed a 28.5% accuracy improvement and an 89.3% reduction in MAPE over models trained on the original monthly data, a difference confirmed statistically via the Wilcoxon signed-rank test. The present study treats SBD as an established preprocessing step and does not replicate that analysis. In the first phase, called Initial Data Generation, the algorithm creates a prior distribution based on the available aggregated information, such as monthly dengue case counts. For example, when monthly totals are available, they are first distributed randomly across the days of that month. The algorithm then adjusts the variance of this distribution to ensure it fits the desired temporal resolution. This process guarantees that the simulated values are non-negative and whole numbers, which is necessary for epidemiological counts, while also fitting a suitable probability distribution [31]. Importantly, the synthetic series can reproduce the original aggregate totals when re-aggregated [27,31].

The second phase, known as Overthrow Correction, deals with problems that arise during the first step, like sudden changes or staircase-like patterns [27,31]. We reduce these issues by redistributing values within limited time frames around the points where these problems occur. This method allows for precise control over the level of smoothing by adjusting tolerance thresholds and setting limits on the number of iterations [27,31].

The third phase, Volume Correction, makes sure that the changes made during smoothing do not alter the original aggregate counts [31]. We achieve this by making small, random adjustments within each aggregation unit so that the total values match exactly with the initial totals. As shown in Table 3, this process maintains the overall case counts while producing realistic downscaled series. This demonstrates that the SBD algorithm is a consistent and statistically sound method for temporal disaggregation in epidemiological studies.

**Table 3:** Comparison of actual vs SDB algorithm generated synthetic data using 2019 DENV data of Bangladesh.

Month	Actual	Initial Distribution	Overthrow Correction	Volume Correction
January	38	38	42	38
February	18	18	13	18
March	17	17	18	17
April	58	58	61	58
May	193	193	300	193
June	1884	1884	2500	1884
July	16253	16253	17617	16253
August	53636	53636	49581	53636
September	16856	16856	18259	16856
October	8143	8143	8419	8143
November	4011	4011	4094	4011
December	1247	1247	1450	1247
**Total**	**102354**	**102354**	**102354**	**102354**

A comparison of the actual dengue case numbers in Bangladesh for 2019 month-by-month with those produced by the SBD algorithm at different stages is shown in the table. At every stage of the process, the overall number of infections stays constant. Following the overthrow correction phase, disparities are noticed in the monthly figures; these anomalies are then fixed in the final volume correction stage, bringing the data back into line with the original aggregated data.

**Fig 3:**
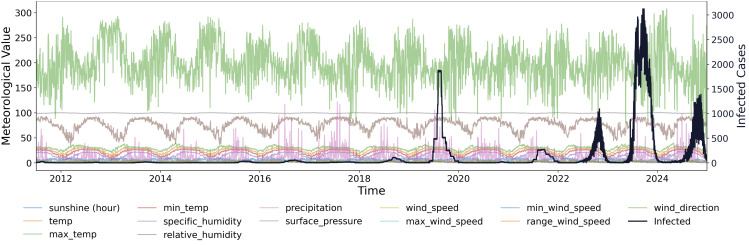
Time series plot of the target variable (DENV infection count) and the thirteen meteorological features. The figure illustrates the times series plot of the DENV infection count (coloured black) and the rest of the meteorological variables (coloured in different colours) using the right hand side and left hand side y-axes respectively. It is evident from the figure that the meteorological variables and the target variables share some degree of common seasonality. The target variable also exhibits major outbreaks in 2019, 2022, and 2023.

For greater clarity about how the method works, the underlying procedure is outlined in the pseudocode in Algorithm (in S1 File). A pictorial representation of the target varaible along with the thirteen meteorological features after SBD downscaling has been presented in Fig 3.

#### Feature engineering.

Feature engineering in time series analysis involves creating new attributes or changing existing ones to improve the predictive performance of deep learning models. This process is very important because it helps the models capture hidden patterns and time-related relationships in the data more effectively. In this study, the feature engineering strategy is organized into three main categories, which are explained in detail below:

- **Lagged features:**

Lagged features capture previous observations of a time series, are important in forecasting as they reflect the temporal construct within a time series data. By using these features, predictive models can draw on past patterns to predict future values, providing insights into how the system changes over time. However, including all possible lags without discretion may lead to unnecessary repetition, increased computational needs, and possibly lower prediction accuracy. Hence, careful selection of the most useful lagged features is key to improve predictive performance.

Before including lagged features from external variables, it’s important to identify those that relate meaningfully to the target variable. In this context, Granger’s causality test is a helpful tool [26,27,32]. Clive Granger first proposed this statistical test to see if past values of one time series can improve predictions of another. It is important to note that the test does not show true causality in the philosophical sense. Instead, it assesses whether one series offers significant predictive value beyond what is already captured by the lagged values of the dependent series itself. The hypotheses are stated as follows:

1. **H**_**0**_**:** Time series *X* does not Granger-cause time series *Y*.
2. **H_1_:** Time series *X* Granger-causes time series *Y*.

Granger’s causality framework is helpful for identifying lagged features that significantly improve forecasting of the target variable. This approach results in simpler models that have better generalization.

To evaluate both short- and long-term effects of weather factors on the target outcome, we considered lags of 1, 2, 3, 7 (weekly), 30 (monthly), 120 (quarterly), 180 (semi-annual), 365 (annual), and 548 days (one-and-a-half years). We applied Granger causality tests across 13 weather variables at these lags. With a significance level of α=0.05, we analyzed a total of 13×9=117 variable-lag combinations. Out of these, 40 produced *p*-values below α, showing their statistical importance in predicting dengue cases. The complete results are detailed in Table in the S2 File. These 40 significant lagged features were then kept as predictors for daily DENV cases.

- **Target derived features:**

Target-derived features are variables created from changes to the target variable itself. These features often include past values of the target series or time-based changes like rolling and expanding window statistics, differencing, seasonal decomposition, and other statistical summaries. Using these features allows models to greatly improve predictive accuracy because they capture historical patterns of the target variable. This boosts the model’s ability to predict future changes. Target-derived features also help make the model’s predictions easier to understand. They show which past values most influence those predictions, giving valuable insights to both experts and decision-makers [33,34].

Among the transformations, rolling and expanding window statistics and differencing effectively address issues related to seasonality and long-term trends in the data. For example, rolling averages smooth out short-term variations, making it easier to see longer-term trends. Differencing removes consistent trends and helps make the series stationary [33].

Seasonal decomposition techniques are also crucial. They break down a time series into separate parts, usually trend, seasonality, and residual noise, which helps improve forecasting accuracy [34]. A key example is the Seasonal Decomposition for Human Occupancy Counting (SD-HOC) model. It uses seasonal-trend decomposition with moving averages to get data ready for regression-based forecasting. This shows how useful seasonal decomposition can be in feature engineering [35]. Adding features from decomposition to predictive models improves accuracy and supports better decision-making in different areas.

The target derived features adopted in the study are as follows:

- **Rolling Window Features:** Mean and standard deviation.
- **Expanding Window Features:** Mean and standard deviation.
- **Seasonal Decomposition Features:** Trend and seasonality.
- **Difference Feature:** The first difference of the target variable.
- **Seasonal Mean:** Acknowledging the annual periodicity of dengue, we took the mean of the target variable for the same day across previous years and applied this to the data.
- **Sparse Indicator:** Due to the high number of zero-valued observations in the DENV infection data, we created a binary indicator to show whether an entry is zero or not. This feature helps machine learning models manage sparsity better and capture the underlying data-generating process.

To capture both short-term and long-term dependencies, we selected daily time windows at several intervals: 2 days, 3 days, 7 days, 90 days (quarterly), 180 days (semiannual), 365 days (annual), 450 days (five quarters), and 540 days (one and a half years).

- **Temporal features:**

Temporal features come directly from the time aspect of time series data. Since the dataset is recorded daily, we extracted the following temporal features: year, month, quarter, week of the year, day, day of the year, day of the week, and a binary indicator that shows whether the observation falls on a weekend (is weekend).

The feature engineering process, especially when creating lagged variables, introduced missing values into the datasets. Most machine learning algorithms do not handle missing data well, so we removed these incomplete observations from the analysis. This step reduces the effective time span of the dataset, but it also increases the feature space. Expanding the dimensionality can improve model performance by offering better representations of the underlying time dynamics.

The table illustrates the transformations applied to the datasets through feature engineering, specifically in terms of time span, number of features, and data points. It is evident from the table that, the time series has decreased by fifteen months, the number of features has increased by almost 6.6 times and the number of data points has increased by almost *six* times.

The final datasets were generated through the feature engineering process illustrated in Fig 1. The changes introduced by this process, in terms of time span, number of features, and data points, are summarized in Table 4. While the feature engineering approach increased the number of features and data points, it was achieved with certain sacrifice of the length of the time series data. This trade-off was necessary to simplify and enhance the subsequent stages of analysis.

**Table 4:** A tabular representation of changes due to feature engineering.

	Feature Engineering	
Attributes	Before	After
Time span	01 Jan 2010 – 31 Dec 2023	03 Jul 2011 – 31 Dec 2023
Number of Features	13	86
Data Points	66469	392590

#### Data preprocessing.

To make the features comparable across different scales, we applied a Min-Max scaling procedure, as shown in Eq (1). This transformation rescales each feature to the interval [0,1] [32]. It prevents variables with larger numerical ranges from having too much influence on the model during training.


$$\begin{array}{c} x_{\text{norm}}=\frac{x-x_{\min}}{x_{\max}-x_{\min}} \end{array}$$ (1)

After normalization, the dataset was split chronologically: the first 90% of observations (July 3, 2011 - August 25, 2023) formed the training set, and the remaining 10% (through December 2024) served as the test set. Random splitting was intentionally avoided to preserve temporal ordering and prevent data leakage.

To prepare the dataset for sequential supervised learning, we reconstructed input-output pairs by reshaping the temporal sequences into suitable training windows. A sliding window approach was then used, allowing the model to use information from the previous 14 days to predict the next 1 day. The model does not natively generate multi-step predictions. The effective nominal forecast lead time is therefore one day. The operationally relevant anticipation window, however, extends to 3–7 days when the meteorological feature structure is considered alongside the model output, as discussed in the Discussion section. This transformation creates a structured dataset, where the dimensionality of the transformed feature space shows the number of samples, the historical window length, and the feature space. The transformed target contains the aligned target values. This preprocessing keeps the temporal dependencies in the data intact and helps the predictive algorithm learn meaningful sequential patterns.

#### Model implementation.

**Fig 4:**
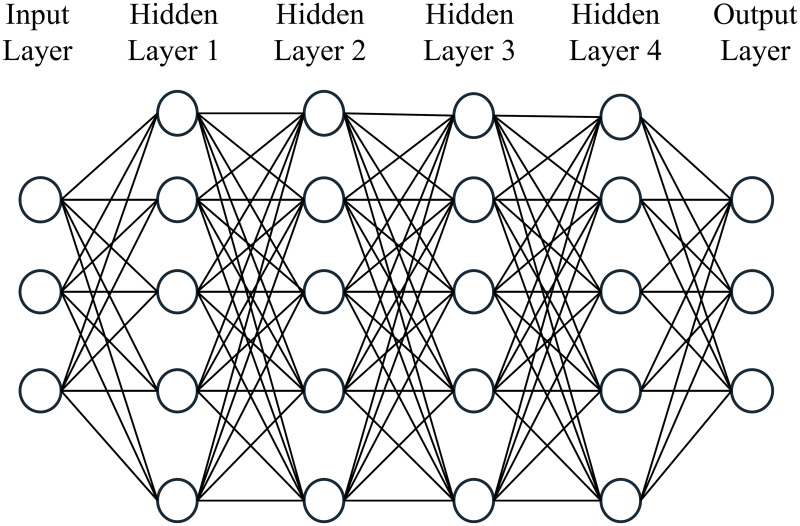
Generalized architecture of the DNN models adopted in the study. The figure depicts the generalized architecture of the DNN adopted in the study. The input is initially passed through four hidden layers of the which in turns return the output.

In recent years, deep neural architectures have become a powerful tool for tackling complex epidemiological challenges, especially those related to infectious diseases that change over time, like DENV infection. In this study, we use a range of machine learning frameworks, including ANN, Recurrent Neural Networks (RNN), and ensemble methods, to model and predict dengue incidence in Bangladesh. These methods are effective for forecasting time series. These models can capture non-linear relationships, time dependencies, and latent structures in epidemiological data that standard statistical methods might miss. By training these models on past dengue case reports, we aim not just for precise predictions. We also want to see how deep learning can uncover hidden time patterns that might provide valuable insights for public health planning and vector control strategies. All the DNN models considered for the experimentations of the study are comprised of four hidden layers except for the ensemble methods where CNN are used as it required an additional pooling layer. A pictorial representation of the generalized model architecture considered the study has been illustrated in Fig 4. A brief discussion on the DNN models considered for the study are as follows:

- **Artificial neural network**

An ANN is a basic type of deep learning model inspired by the structure and function of the human brain. It includes a series of connected units called neurons, organized into different layers. Information enters the system through the input layer, changes across one or more hidden layers, and concludes with a prediction at the output layer. Each neuron processes weighted inputs from the previous layer, applies a transformation, and sends the result to the next layer. The hidden layers are essential for identifying non-linear relationships and pulling out key features from the raw data.

Formally, in this study the input vector at each prediction step is $𝐱=[x_{1},x_{2},\ldots ,x_{86}{]}^{⊤}$, where the 86 components correspond to the engineered feature set: 40 Granger-significant lagged meteorological variables (e.g., surface_pressure_lag3, max_wind_speed_lag7, precipitation_lag7), target-derived rolling and expanding statistics, seasonal decomposition components, and temporal indicators. The synaptic weights $w_{1},w_{2},\ldots ,w_{86}$ are initialized randomly and learned by minimizing mean squared error on the training set via the Adam optimizer. The scalar output *y* is the predicted normalized dengue case count for day *t* + 1, subsequently inverse-transformed to the original case scale. The weighted sum (as illustrated in (2)) calcula*t*ed at a neuron can be described as:


$$\begin{array}{c} u=\sum_{j=1}^{n}w_{j}x_{j}, \end{array}$$ (2)

where *u* indicates the net input. The neuron then applies a non-linear activation function ϕ to this sum, with an added bias term *b*, to produce the final output:


$$\begin{array}{c} y=\phi (u+b). \end{array}$$ (3)

In this context, ϕ acts as the activation function, controlling the network’s ability to map non-linear relationships, while *b* adds flexibility to the decision boundary.

- **Recurrent neural network**

RNNs build on the traditional feed-forward neural architecture to handle sequential and time-related data, such as time series, natural language, and speech signals. Unlike classical ANNs, RNNs have feedback loops in their design. This feature allows them to keep contextual information from previous time steps. Because of this, RNNs have a form of memory represented as recurrent hidden states, which helps model relationships over time across sequences.

Formally, In this study, the input sequence is $𝐱=(x_{1},x_{2},\ldots ,x_{14})$, where *T* = 14 days is the sliding window length and each $x_{t}\in {\mathbb{R}}^{86}$ is the full engineered feature vector for day *t*. The hidden state $h_{t}$ encodes the model’s learned summary of meteorological and epidemiological conditions over the preceding 14 days. Recurrent weigh*t* matrices $W_{xh}$ and $W_{hh}$ are learn ed by backpropagation through time. For LSTM specifically, the forget gate governs retention of cumulative climate signals such as sustained rainfall or persistent low surface pressure across the window, which is epidemiologically relevant given the lagged breeding-cycle dynamics documented in the feature importance analysis. The hidden state update (as illustrated in (4)) is given by:


$$\begin{array}{c} h_{t}=\sigma (W_{xh}x_{t}+W_{hh}h_{t-1}+b_{t}), \end{array}$$ (4)

where σ is a non-linear activation function. This is usually the logistic sigmoid, hyperbolic tangent, or rectified linear unit. The matrix $W_{xh}$ relates to the input, $W_{hh}$ is the recurrent weight matrix, and $b_{t}$ is the bias term. The output at each time step (as illustrated in (5)) is calculated as:


$$\begin{array}{c} y_{t}=W_{hy}h_{t}, \end{array}$$ (5)

where $W_{hy}$ is the output weight matrix.

Several approaches have been created to address issues like vanishing and exploding gradients and to improve long-term dependency modeling. One notable variant is the Long Short-Term Memory (LSTM) architecture. LSTMs use specialized memory cells called *cell states*. These cells keep information over longer time periods. Three gates control the behavior of the cell: the *forget gate* decides which past information to discard, the *input gate* manages the addition of new information, and the *output gate* regulates how much the cell state contributes to the current output [36–38].

The Bidirectional LSTM (BiLSTM) is a natural extension that includes two parallel LSTM networks. One processes the input sequence forward, and the other processes it in reverse. This setup improves the model’s ability to represent information by using both past and future context [36,39].

The Gated Recurrent Unit (GRU) provides a simplified alternative to LSTMs. By combining the input and forget gates into a single *update gate*, GRUs allow for efficient training while still performing well on tasks that involve sequential dependencies. This design makes it easier to model both short- and long-range patterns with less computational effort, making GRUs a good choice for applications like natural language processing, machine translation, and time series forecasting [40]. Its bidirectional version, the BiGRU, adds to this capability by combining forward and backward processing streams.

Recently, attention mechanisms have been added to support recurrent models, leading to structures like LSTM-ATT and GRU with Attention (GRU-ATT). These mechanisms assign different importance to elements of the input sequence based on context, which helps the model focus on the most important features. By improving the handling of long-term dependencies, attention-based RNNs significantly boost performance in complex prediction tasks, including disease forecasting [41].

- **Ensemble method**

**Fig 5:**
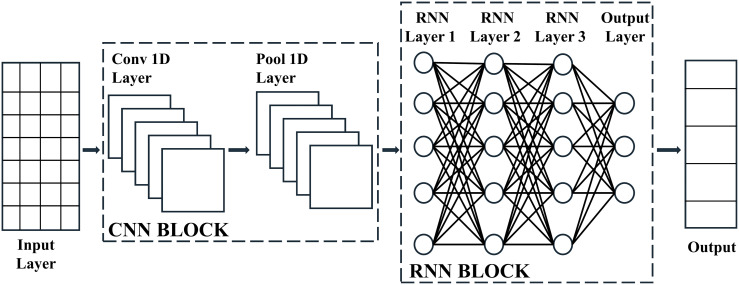
Generalized architecture of the ensemble methods adopted in the study. The figure depicts the generalized architecture of the CNN-RNN ensemble adopted in the study. The input is initially passed through a CNN block followed by three RNN layers which in turns return the output.

In this framework, we use several hybrid deep learning models, including CNN-LSTM, CNN-BiLSTM, and CNN-GRU, to evaluate and improve the predictive accuracy of dengue cases in Bangladesh as generalized architecture of which has been illustrated in Fig 5. These combined architectures leverage the feature extraction capability of CNN along with the sequential modeling strengths of recurrent architectures like LSTMs, BiLSTMs, and GRUs.

CNNs are specialized neural networks designed to work with data that has an innate grid-like structure. This includes one-dimensional (1D) time-series signals, two-dimensional (2D) images, and even three-dimensional (3D) volumetric data [42]. Their structure features three key components: convolutional layers, which learn local patterns through kernel operations; pooling layers, which lower dimensionality while keeping important features; and fully connected layers, which enable classification or regression at the end stage. Nonlinear activation functions in these layers help approximate complex mappings [43–45].

When combined with recurrent models, CNNs serve as effective feature extractors, pulling useful representations from raw, often noisy input data. The recurrent layers LSTM, BiLSTM, or GRU, then capture time-related dependencies and sequence dynamics from these features. Specifically, LSTM and GRU networks address the vanishing gradient problem and maintain long-range dependencies. The bidirectional variant, BiLSTM adds to the richness by including both forward and backward context. These hybrid architectures perform better than single-model approaches because they use complementary strengths. CNNs filter and abstract features effectively. Recurrent models, on the other hand, maintain and use sequential patterns that are crucial for time-series forecasting [46].

#### Model hyperparameter tuning.

Hyperparameter optimization is an important step in creating effective DNNs, especially in complex fields like public health informatics. Traditional grid or random search methods are simple to understand, but they often come with high computational costs [47]. These also do not fully use the structure of the objective function, which impacts model performance [48]. In contrast, Bayesian optimization offers a more organized and efficient way to conduct the search [48]. It employs probabilistic models to direct the search toward areas of the hyperparameter space that are likely to boost performance [48].

In this study, we used Bayesian optimization to fine-tune the key hyperparameters of the DNN. We set a limit of 200 optimization trials. This allowed us to explore nonlinear interactions among hyperparameters without overloading computation. To keep a balance between exploration and exploitation, we designated 10% of the trials (20 evaluations) as initial random points. This initial phase has two main purposes. It ensures a wide variety of hyperparameter samples, which reduces bias in the surrogate model. It also improves the stability of the following Gaussian process approximations or other surrogate methods like Tree-structured Parzen Estimators [49].

After this warm-up phase, the optimization process refined its search strategy. Each trial used the average and variance from the surrogate. Acquisition functions like Expected Improvement (EI) or Upper Confidence Bound (UCB) helped balance the need to explore new options and make the most of the best choices [50]. This ongoing improvement helped us understand the hyperparameter landscape better, leading to faster results than brute-force methods [48,50].

The careful design of this optimization framework is important. Models in this area often influence decisions that affect public health. Therefore, it is vital to ensure both accuracy and generalizability of the DNNs. By limiting the optimization to a well-planned number of trials and properly organizing the initialization, we achieved efficiency and improved reproducibility. This consistency is key in translational research, where policy decisions depend on clear and repeatable analysis methods.

Table 5 depicts the hyperparameter space used for Bayesian optimization in the study. Tables in S3 File summarize the detailed best performing topologies of each architecture.

**Table 5:** Hyperparameter space considered for Bayesian optimization.

Tuned Hyperparameter	Search Space	Description
filters	{32, 64, 96, 128}	Number of convolutional filters in the initial Conv1D layer.
kernel_size	{2, 3, 4, 5}	Temporal receptive field of the convolutional layer.
activation_cnn	{ReLU, Tanh, ELU}	Nonlinear activation function for convolutional output.
pool_size	{2, 3, 4}	Subsampling factor in max-pooling layer.
units_{1–3/4}	{32–256, step = 32}	Fully connected hidden units/Hidden units per RNN layer.
activation_{1–3/4}	{ReLU, Tanh, ELU}	Activation function within ANN/RNN units.
dropout_rate_{1–3/4}	[0.0, 0.25]	Fraction of units dropped in recurrent layers.
learning_rate	[1e-5, 1e-2] (log scale)	Initial learning rate for Adam optimizer.

The table illustrates the hyperparamaeter space considered for Bayesian optimization. The first four hyperparameters (filters, kernel_size, activation_cnn, and pool_size) are for the CNN blocks of the ensemble architecture. The rest of the hyperparamters are for the ANN/RNN layers. The ‘3/4’ implies three or four hidden layers considered usual or ensemble architecture as illusterated in Fig 5 and 4 respectively.

We proceed by fitting the hyperparameter-tuned models on the training dataset and then evaluating their performance on the test set to identify the optimal model. The metrics employed for this evaluation are:

- Root Mean Square Error (RMSE) $=\sqrt{\frac{1}{n}\sum_{t=1}^{n}{\left(y_{t}-{\hat{y}}_{t}\right)}^{2}}$
- Mean Absolute Error (MAE) $=\frac{1}{n}\sum_{t=1}^{n}\left|y_{t}-{\hat{y}}_{t}\right|$
- Mean Absolute Percentage Error (MAPE) $=\frac{100\%}{n}\sum_{t=1}^{n}\left|\frac{y_{t}-{\hat{y}}_{t}}{y_{t}}\right|$
- Accuracy/Coefficient of Determination (*R*^2^) $=1-\frac{\sum_{t=1}^{n}{\left(y_{t}-{\hat{y}}_{t}\right)}^{2}}{\sum_{t=1}^{n}{\left(y_{t}-\bar{y}\right)}^{2}}$

Where, $y_{t}$, ${\hat{y}}_{t}$ are the actual and the predicted values at any time, *t*.

#### Feature selection.

Redundant features are variables that do not provide much new information beyond what others already capture. They can distort prediction accuracy, increase model complexity, and lead to overfitting, which occurs when a model performs well on training data but poorly on new data. Removing redundancy is essential. It simplifies models, makes them easier to understand, reduces multicollinearity, and lowers computational needs. Feature selection, in general, is the process of reducing dimensionality while keeping explanatory and predictive accuracy [51]. Checking every possible subset, which is $(2^{N})-1$ for *N* features, is not practical (NP-hard). Therefore, effective methods use heuristic or sequential strategies that strike a balance between accuracy and manageability.

This study uses the Sequential Squeeze Feature Selection (SSFS) algorithm as a wrapper feature selection method for DNN models [26,27]. It was previously applied to ML models. SSFS combines top-down (backward elimination) and bottom-up (forward inclusion) approaches in one repeating process. This design avoids the nesting and backtracking problems seen in earlier methods. At its center, SSFS operates on a custom accuracy-focused objective function (as illustrated in Eq (6)):


$$\begin{array}{c} z_{k}(f_{i})=\left\{ \begin{array}{cc} A_{F_{k}}-A_{F_{k}-\{f_{i}\}}, & f_{i}\in F_{k}\;\text{(truncation case)}\\ A_{F_{k}\cup \{f_{i}\}}-A_{F_{k}}, & f_{i}\in E_{k}\;\text{(inclusion case)} \end{array}\right. \end{array}$$ (6)

Here, $A_{F_{k}}$ denotes model accuracy using the feature subset $F_{k}$, while $E_{k}$ is the set of eliminated features at iteration *k*. A feature is deemed redundant if its marginal contribution falls below a threshold ϵ>0:


$$\begin{array}{c} z_{k}(f_{i})<\epsilon ,\;\exists k\in {\mathbb{N}}_{n(F)-1}. \end{array}$$ (7)

The SSFS algorithm allows features to move back and forth between the active set $F_{k}$ and the eliminated set $E_{k}$. This lets us revisit features that might have been removed too soon. Unlike traditional sequential methods, SSFS does not need a set number of features for the final subset ahead of time. Instead, the stopping rule is based on data. It is determined by the objective function and the threshold ϵ.

In practice, SSFS operates in two stages per iteration. The first stage is backward elimination. In this stage, features that contribute little are removed. The second stage is forward recovery. Here, we reconsider features that we previously removed if they improve accuracy. This process goes on until all remaining features exceed the redundancy threshold. The final results are the retained feature set *F* and the eliminated set *E*.

The framework also offers a straightforward measure of feature contribution to accuracy as stated in Eq (8).


$$\begin{array}{c} A\_\%(f_{i})=\frac{z_{N}(f_{i})\times 100}{A_{F_{N}}\times \sum_{f_{j}\in F_{N}}z_{N}(f_{j})}\;\%, \end{array}$$ (8)

where $F_{N}=F$ denotes the final subset after *N* iterations. This quantity expresses each feature’s marginal accuracy contribution as a proportion of the total accuracy explained by the retained feature subset $F_{N}$. Concretely, $z_{N}(f_{i})$ is the final-iteration marginal contribution of feature $f_{i}$, the accuracy drop when it is added/removed from $F_{N}$ and the denominator normalises these contributions so that all retained features sum to 100%. This derivation is fully self-contained within the SSFS framework [26]. This quantity offers a transparent link between each feature and the model’s overall predictive performance, enhancing interpretability alongside efficiency.

SSFS advances sequential feature selection by introducing a theoretically grounded, accuracy-driven, and bidirectional search process. It balances parsimony with fidelity, mitigates longstanding issues such as nesting, and equips researchers with a robust tool for feature selection in high-dimensional settings. For ease of implementation, detailed pseudocode of the SSFS algorithm has been presented in the S4 File.

## Results

### Learning curve

**Fig 6:**
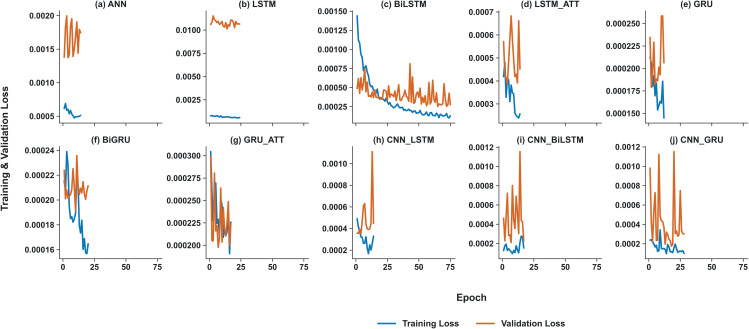
Faceted learning curves of training and validation loss (MSE) for all ten deep neural network architectures evaluated in the study. Each panel displays epoch-level mean squared error for the training set (blue) and the chronological validation split (orange) under identical training conditions: best Bayesian-optimised hyperparameters, early stopping with patience of 200 epochs, batch size of 128, and a 10% chronological validation split.

As per the benchmarking literature [52], All ten architectures converge cleanly without divergence between training and validation loss, confirming stable optimization as illustrated in Fig 6. The ANN converges fastest and to the lowest validation loss. Recurrent models, particularly LSTM and LSTM-ATT, show persistent train-validation gaps and higher terminal validation loss consistent with mild over-fitting induced by redundant temporal parameterization on a feature space that has already been temporally structured.

Structural patterns emerge from reading the ten panels together. Architectures whose training loss reaches near-zero early while validation loss remains an order of magnitude higher (most clearly LSTM (panel b)) are exhibiting feature-level over-fitting: the recurrent hidden state has memorized training-specific lag structures that the 86-feature engineering pipeline has already made explicit, providing no generalization benefit from doing so twice. Architectures with volatile validation loss despite stable training loss (BiLSTM (panel c), CNN-LSTM (panel h)) indicate that the sequential processing layers are introducing optimization instability when confronted with pre-encoded temporal structure, producing inconsistent gradient estimates across validation batches. Architectures where training and validation loss converge to similar absolute values(GRU (panel e), GRU-ATT (panel g), and late-stage CNN-BiLSTM (panel i)) indicate that the gating or hybrid mechanism has found a representation sufficiently compact to generalize, though none fully close the gap to the ANN’s combination of stable convergence, moderate absolute loss level, and non-widening train-val separation. The ANN’s advantage is therefore not that it achieves the lowest absolute training or validation loss (GRU approaches it on both) but that it does so with the most consistent and non-volatile trajectory across the full training process, a property that compounds into superior and more predictable performance when the test window shifts to unseen outbreak magnitudes.

### Rolling train test split performance analysis

**Fig 7:**
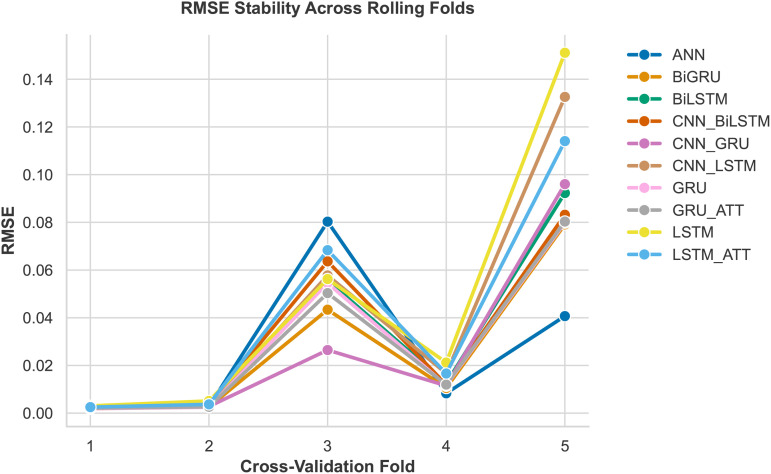
Fold-by-fold RMSE stability across five rolling cross-validation folds for all ten deep learning architectures. Each line traces the RMSE of a given architecture on the held-out test window of each expanding fold, with fold number on the x-axis representing chronologically advancing test periods. Folds 1 and 2 correspond to test windows covering the pre-2019 low-transmission baseline period; fold 3 introduces the first major outbreak cycle into the test set; fold 4 covers the post-peak recovery and 2022 resurgence window; fold 5 encompasses the 2023 record epidemic and the 2024 period.

To address whether the ANN’s superiority observed in the single 90/10 train-test split generalizes across different temporal windows and outbreak cycles, we evaluated all ten architectures under a five-fold expanding-window time-series cross-validation scheme. The distribution of RMSE across folds is presented in Fig 7.

The per-fold RMSE plot reveals a structured pattern that carries epidemiological content beyond the aggregate metrics. On folds 1 and 2, all ten architectures converge to near-zero RMSE, a finding that reflects the low-transmission baseline period these early folds cover, where case counts are sparse and the prediction task is comparatively straightforward regardless of architecture. The absence of differentiation at folds 1 and 2 is itself informative: no architecture holds a structural advantage when the target series is near-flat, which is consistent with the view that architectural differences matter primarily under outbreak conditions.

Fold 3 introduces a shared RMSE spike across all models, which corresponds to the first test window containing a major outbreak cycle. Here, every architecture struggles relative to its fold 1–2 performance, confirming that rapid epidemic acceleration is the genuinely hard forecasting problem in this dataset, not the inter-epidemic baseline. Notably, the ANN’s fold 3 spike (∼0.081) is the highest among all models at that fold, marginally exceeding recurrent architectures such as CNN-LSTM (∼0.065) and BiLSTM (∼0.065). This is the one fold where the ANN’s parallel feature-processing architecture does not hold an unambiguous advantage. Recurrent models, which propagate hidden state through the ascending sequence, may carry marginal advantage at this inflection point.

However, fold 4 sees an immediate and sharp recovery for the ANN, returning to near-zero RMSE (∼0.018), the lowest of all models at that fold. This recovery is faster and more complete than for any recurrent architecture. It suggests that once the epidemic peak passes and the descending phase begins, a trajectory better represented in the lagged feature history, the ANN reasserts its advantage quickly. Recurrent models show a more gradual recovery, consistent with their hidden states retaining residual influence from the high-case-count peak, which acts as a form of inertia during the descending phase.

Fold 5 is the most discriminating fold in the entire cross-validation. All recurrent models experience a second and substantially larger RMSE escalation at fold 5, with LSTM reaching the highest error of any model at any fold (∼0.150). LSTM-ATT follows at approximately 0.115. BiGRU (∼0.085), CNN-LSTM (∼0.133), BiLSTM (∼0.092), GRU (∼0.081), and GRU-ATT (∼0.081) all show marked deterioration. CNN-GRU is the most stable recurrent architecture, with a fold 5 RMSE below 0.10 and one of the tightest trajectories throughout folds 3–5. The ANN, by contrast, reaches only ∼0.041 at fold 5, less than a third of LSTM’s error and lower than every recurrent architecture except CNN-GRU in some metrics. This divergence at fold 5 is the strongest empirical statement the cross-validation analysis makes: as test windows move into the 2023–2024 period, with its record outbreak magnitude, recurrent models degrade substantially while the ANN degrades modestly. The likely mechanism is that recurrent hidden states, optimised during training on earlier outbreak patterns, do not generalise well to the scale of 2023 case counts, whereas the ANN relies on the same pre-engineered lagged meteorological and target-derived features that remain structurally valid regardless of absolute case magnitude.

The RMSE stability plot across five chronological rolling folds reveals that ANN maintains the most consistent error profile. LSTM exhibits severe instability in fold 5, spiking to RMSE ≈ 0.15, the highest of any model at any fold.

#### Results of the performance of the models.

**Fig 8:**
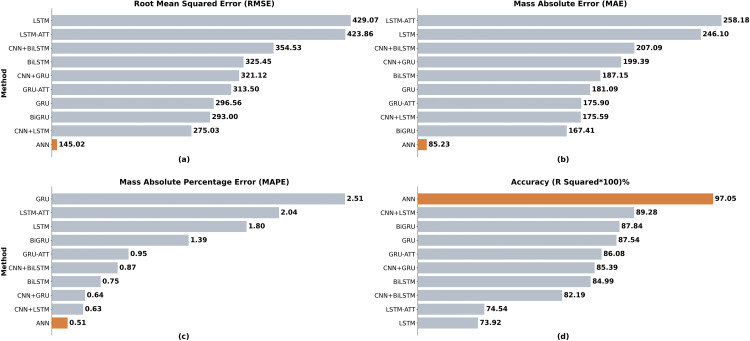
Metric comparison of the best architecture of different DNNs considered for the study. The figure depicts the performance metric comparison of the best architecture of each type of models considered for the study. The ANN outperforms all other architectures on this dataset. The gap between ANN and recurrent or attention-based models reflects the interaction between the depth of the feature engineering pipeline and each architecture’s inductive bias, rather than a general superiority of feed forward networks over recurrent ones.

The evaluation of different deep learning models based on Fig 8 reveals significant variation in their predictive performance, highlighting the strengths and limitations of each architecture in forecasting dengue incidence in Bangladesh.

The comparison of model performance shows that the ANN consistently outperforms all other architectures. It reaches the highest accuracy of 97.05% and has the lowest error metrics, with RMSE at 145.02, MAPE at 0.51, and MAE at 85.23. This demonstrates both precision and reliability in its predictions. Recurrent models, especially LSTM and LSTM-ATT, have lower accuracy and higher errors. This suggests a possible mismatch between model complexity and data structure. Models based on GRUs, including bidirectional ones, perform slightly better but still do not match the ANN. CNN-LSTM is a hybrid convolutional recurrent architecture that works very well. It achieves an accuracy of 89.28% and an RMSE of 275.03. It likely performs better because it can extract features effectively, while CNN-BiLSTM does not perform as well. These results should not be read as a general indictment of recurrent or attention-based architectures. LSTMs, GRUs, and their bidirectional and attention-augmented variants are well-suited to raw or minimally processed sequential data, where the model must learn temporal dependencies from scratch through its internal gating mechanisms. The present dataset is different in a specific way: the feature engineering pipeline has already made those temporal dependencies explicit. Granger-selected lagged meteorological features, rolling window statistics across varying time horizons, seasonal decomposition components, and first-difference terms collectively pre-encode the lag structure that a recurrent model would otherwise have to discover internally. Once that structure is handed to every model as input, the recurrent hidden state provides less marginal information and its sequential processing bottleneck becomes a liability relative to a feed forward network that can weight all 86 features simultaneously across the full 14-day window.

This interaction between feature engineering depth and architectural inductive bias is documented in the broader time series literature [53–56]. The present finding extends this pattern to dengue epidemiology: the ANN’s advantage is dataset-conditional, not architectural. On raw or coarsely aggregated dengue data with minimal feature engineering, recurrent models would likely recover a meaningful performance edge. Researchers applying these methods to different surveillance contexts (shorter time series, monthly resolution, or fewer meteorological covariates) should not assume the ANN will dominate, and should evaluate recurrent architectures on their own data accordingly.

### Train-test fit assessment

**Fig 9:**
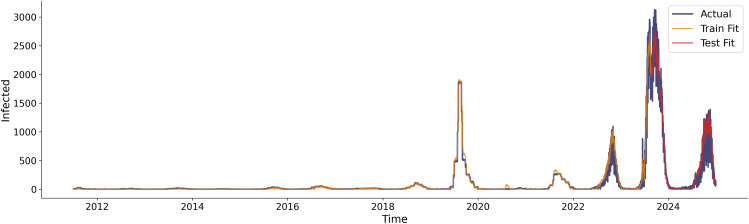
Train test fit of the best performing model, ANN. The figure depicts the train test fit of the best performing ANN architecture. It is evident from the figure as well as the metric evaluation of the ANN architecture that the model not only fits well on the training data but also generalizes better on the unseen test data.

Following the quantitative evaluation in the previous section, where the ANN achieved an accuracy of 97.05%, MAE of 85.23, MAPE of 0.51, and RMSE of 145.02, we will now look at a qualitative assessment of its fit across the training and test data. Fig 9 shows the model’s fitted values alongside the actual dengue incidence over time.

The results demonstrate the ANN’s strong ability to mimic the patterns of dengue transmission. In the training set, the predicted curve closely follows the real trajectory, capturing both the timing and strength of the outbreak waves with impressive accuracy. The model tracks the large epidemic peaks as well as the smaller fluctuations between epidemics, which can be difficult to predict due to their random nature. This indicates that the ANN has effectively learned the complex time relationships that define dengue spread in areas where it is common.

**Fig 10:**
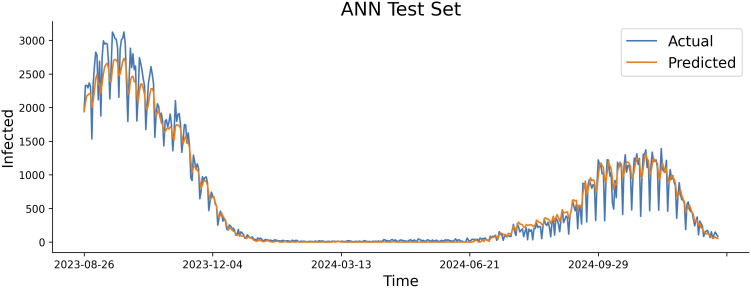
Test fit of the best performing model, ANN. The figure depicts the test fit of the best performing ANN architecture. It is evident from the figure as well as the metric evaluation of the ANN architecture that the model generalizes better on the unseen test data.

Equally important, the test results show the model’s ability to generalize as better illustrated in Fig 10. Even though this data was not used for training, it reveals a strong match between the observed and predicted dengue cases. The ANN performed well in predicting both the timing and size of the successive peaks, with only slight differences during the rapid rise and fall of outbreak cycles. These differences, while noticeable at high case counts, are within acceptable limits for epidemiological data and likely reflect the natural complexity of sudden epidemic changes rather than any flaws in the model.

### Feature importance

**Fig 11:**
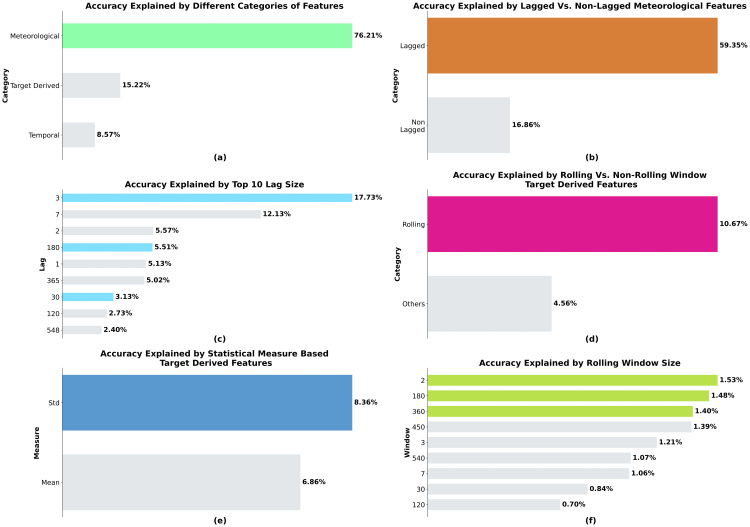
Comparative analysis of feature importance and percentage of accuracy explained by variables. The figure depicts the comparative analysis of features importance of different class of features considered for the study.

A clear understanding of feature importance is key in predictive modeling, especially for forecasting a complex phenomenon like dengue transmission. Figs 11 through 16 provide a detailed analysis of how various types of variables: meteorological, lagged, target-derived, and temporal contribute to the success of predictive models.

**Fig 12:**
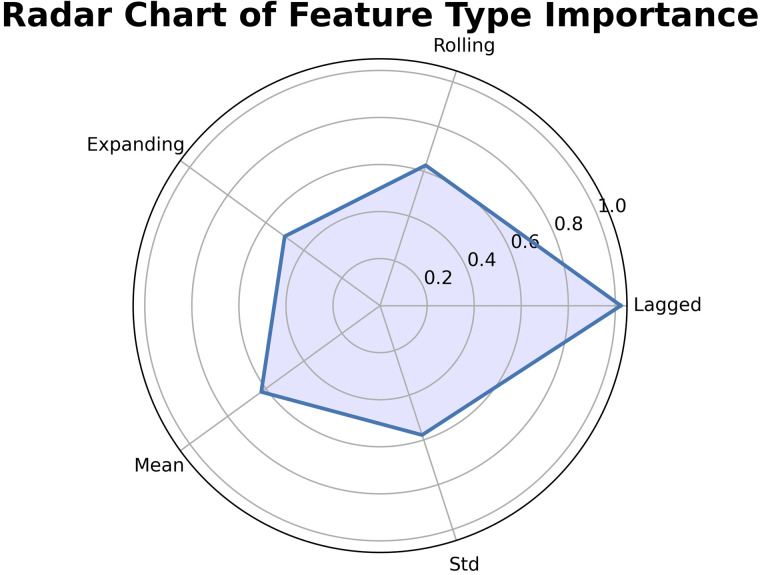
Radar plot of average importance of different category of features. The figure depicts the comparative analysis of average features feature importance of different class of features considered for the study. It is evident that the lagged features on average hone better explanatory power of DENV infection in Bangladesh over other categories of features which supports the lagged effect of meteorological factors on DENV propagation.

**Fig 13:**
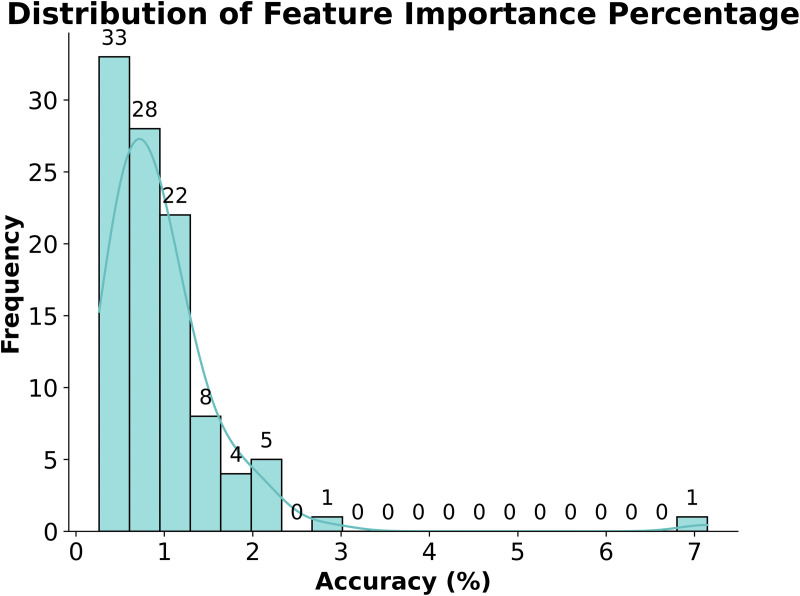
Histogram of accuracy (%). The figure illustrates the frequency distribution of varability explained features. It is evident that majority of features has explanatory power in the vicinity of one, while some of them around two and only feature has explanatory power around seven percent.

**Fig 14:**
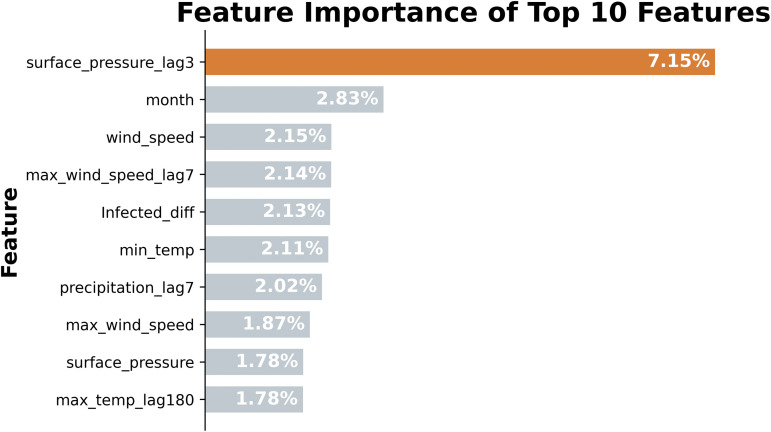
Feature importance of top ten features. The figure illustrates the top ten features in terms of accuracy (%) explained each feature. It is evident that the surface pressure at a lag of 3 days is the most dominant features. Of the top ten features, 80% are meteorological features and it’s lagged version, 10% is target derived feature, and the remaining 10% is temporal features. This emphasizes the importance of meteorological features in modeling DENV transmission.

**Fig 15:**
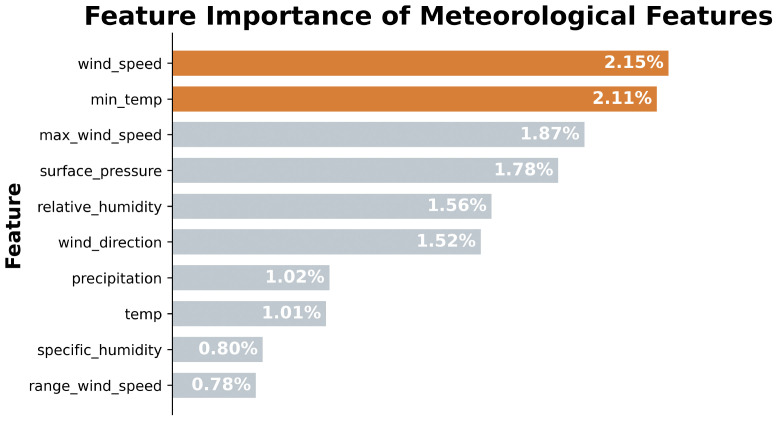
Feature importance of top ten meteorological features. The figure illustrates the top ten meteorological features in terms of accuracy (%) explained each meteorological feature. It is evident that the wind speed and minimum temperature are two of the most dominant metrological features to predict DENV transmission.

**Fig 16:**
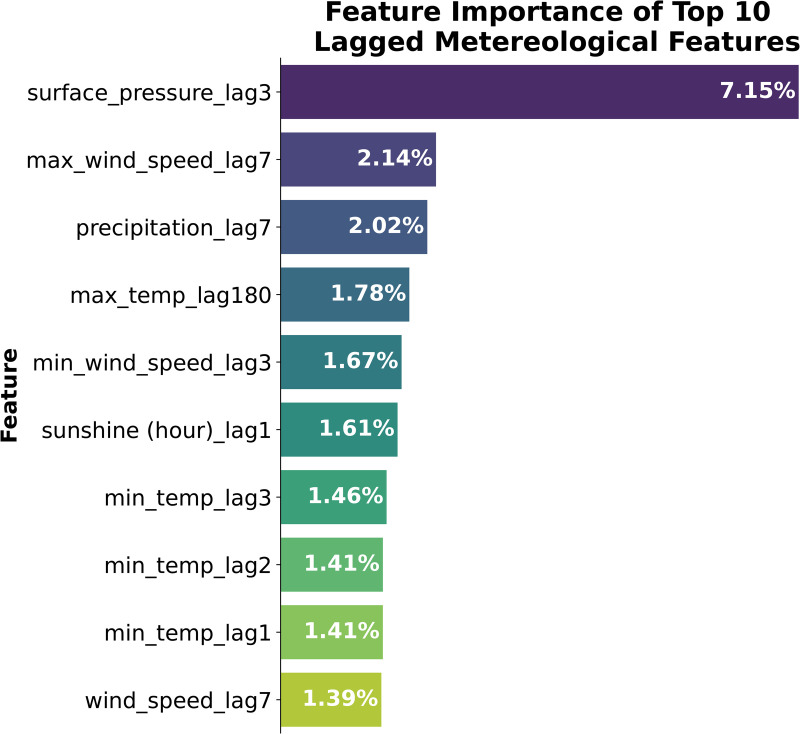
Feature importance of top ten lagged meteorological features. The figure illustrates the top ten lagged meteorological features in terms of accuracy (%) explained each feature. It is evident that the surface pressure at a lag of 3 days is the most dominant features followed by maximum wind speed and precipitation both at lag 7. Observing the lags, it is evident that the impact of short term is most dominant and only long term lag impact is observed for maximum temperature.

Fig 11 presents a multi-faceted view of feature importance, showing six different but related angles on how various variable classes affect the accuracy of dengue forecasting models. By examining these sub-figures one by one, a clear hierarchy appears, ranging from broad feature categories to specific lagged meteorological elements and detailed target-derived transformations.

Fig 11A outlines the basic hierarchy among categories. Meteorological features stand out as the strongest predictors, accounting for 76.21% of model accuracy. In contrast, target-derived features contribute 15.22%, while temporal features make up just 8.57%. This shows that dengue dynamics in Bangladesh largely depend on climate, with autoregressive patterns and calendar-based factors playing a minor role.

Fig 11B separates meteorological predictors into lagged and non-lagged groups. Lagged features account for 59.35% of model accuracy, nearly four times the contribution of non-lagged features at 16.86%. This gap is not surprising once it is interpreted in light of *Ae. aegypti* biology. The species completes its gonotrophic cycle blood meal to egg laying in roughly 3–4 days at 28–30°C [57,58]. A rainfall event therefore does not translate into elevated adult densities immediately; it first fills oviposition containers, sustains larval development over 5–8 days at ambient Bangladesh temperatures, and only then releases newly emerged adults into the biting population. Lagged predictors capture exactly this sequence. Non-lagged weather variables, however precise, describe conditions that have not yet had time to propagate through the vector life cycle into observable case counts.

Fig 11C shows which lag lengths matter most. The 3-day lag (17.73%) and 7-day lag (12.13%) dominate, while lags of 120 and 548 days contribute under 2.5% each. The 3-day signal corresponds to the window in which adult female *Aedes* respond behaviorally to microclimatic changes flight activity, host-seeking range, and resting-site selection all shift within 48–72 hours of pressure or humidity perturbation [59]. The 7-day lag aligns with the time from container flooding to the emergence of a new adult cohort under warm-season conditions. Together, these two lag windows represent the biological front end of the transmission chain: the period between environmental trigger and infectious vector. Long-lag predictors (>90 days) likely capture residual inter-annual climate variation, including ENSO-related anomalies, but their lower explanatory weight confirms that outbreak forecasting in Bangladesh operates mainly on short ecological timescales. Biologically, this lag dominance is expected but requires careful reading. The Aedes aegypti life cycle spans 8–14 days under Bangladesh’s tropical conditions, and the extrinsic incubation period of DENV in the vector adds a further 8–12 days [60]. Short lags of 3 and 7 days therefore do not imply direct causation within that window. Rather, surface pressure and precipitation at these lags most plausibly act as triggers on a mosquito population already mid-cycle, accelerating larval habitat formation rather than initiating new transmission chains.

The focus then shifts to target-derived features in Fig 11D. Here, rolling window features (10.67%) are much more informative than their non-rolling counterparts (4.56%). This indicates that smoothing techniques, which emphasize short-term trends while reducing noise, improve predictive power more than static or decomposed transformations can provide.

Fig 11E compares statistical measures within target-derived features. Rolling standard deviation (8.36%) outperforms rolling mean (6.86%). This is not a minor technical detail. In outbreak epidemiology, rising variance in incidence time series is a recognized precursor to epidemic transition, a phenomenon described in the early-warning indicators literature as “critical slowing down” [61,62]. When a system approaches a tipping point, it recovers more slowly from small perturbations, producing visibly increased fluctuation around the mean before the mean itself shifts sharply. Rolling standard deviations computed over short windows effectively detect this instability phase. Averages, by contrast, lag the transition; they reflect where the epidemic has been, not where it is heading. The model’s preference for variance over mean is therefore consistent with the view that dengue outbreaks in Bangladesh are threshold events, not gradual accumulations.

Fig 11F shows that the most useful rolling window sizes are very short (2 days, 1.53%) and seasonally long (180–450 days, approximately 1.4%), while intermediate windows of 7 and 30 days contribute least. This bimodal pattern has a biological reading. The 2-day window captures day-to-day stochastic noise in reporting and transmission essentially, the acute signal of whether today’s case count differs from yesterday’s in a way that suggests acceleration. The 180–450 day window captures the broad seasonal envelope of *Ae. aegypti* population dynamics, including the monsoon-driven surge and dry-season collapse that repeat each year with some inter-annual variation. The 7- and 30-day intermediate windows sit in an informational gap: they are too long to capture acute transmission bursts and too short to capture the seasonal cycle, so they add little that the flanking windows do not already provide.

These six sub-figures in Fig 11 depicts a clear findings. Meteorological variables are central to dengue prediction, with lagged features, especially short-term lags of meteorological variables being the most important contributors. Target-derived features offer additional value, especially when designed to capture variability rather than averages and targeted at very short or seasonal time frames. Temporal calendar features, by contrast, are less significant. This layered hierarchy highlights an important takeaway: dengue forecasting in Bangladesh is primarily driven by climate, influenced by time delays, and sensitive to both short-term changes and long-term seasonal patterns.

While each category adds some predictive value, it is clear that meteorological and lagged features dominate the importance landscape. This is shown more clearly in Fig 12, where the radar plot displays lagged features as consistently having higher average explanatory power. This highlights the known delays in how weather affects dengue spread. These findings support current theories in epidemiology. Mosquito breeding cycles, viral incubation times in mosquitoes, and human-mosquito interactions all happen with natural time delays [2].

The data in Fig 13 supports this argument. The histogram of accuracy contributions by feature shows a clear right-skewed pattern. Most features cluster around low explanatory values of 1–2%, with only a few making a significant impact. This indicates that while many features contribute to the model’s robustness, the main predictive strength depends heavily on a small number of key predictors.

Fig 14 reinforces this by ranking the ten most influential features. Notably, surface pressure with a 3-day lag is the top predictor. The fact that 80% of the ten most important features are meteorological (or their lagged versions), with only one coming from target-derived transformations and one from temporal indicators, highlights the significant role of environmental factors in shaping dengue patterns in Bangladesh.

Figs 15 and 16 provide more insight by focusing exclusively on meteorological and lagged meteorological features. Identifying wind speed and minimum temperature as key immediate predictors supports earlier field studies that link these factors to mosquito survival and viral replication. Additionally, the top predictors: surface pressure (3-day lag), maximum wind speed (7-day lag), and precipitation (7-day lag) show that short-term lags are more predictive than long ones. The only long-lagged variable, maximum temperature, seems to affect transmission cycles only in certain situations, likely because of cumulative ecological changes.

The dynamics of dengue transmission in Bangladesh depend on a complex interaction of immediate and lagged meteorological factors, with a few key variables having a disproportionate impact on predictive accuracy. The main points from this detailed analysis of feature importance are:

1. Meteorological features account for 76.21% of model accuracy. Dengue in Bangladesh is, at its core, a climate-driven disease, the epidemiological signal is inseparable from the atmospheric one.
2. Lagged meteorological variables (59.35%) outperform non-lagged ones (16.86%) by a factor of roughly four. This gap reflects the biology: weather does not act instantaneously on case counts. It acts through the *Ae. aegypti* life cycle (filling breeding containers, sustaining larval development at 5–8 days, and driving adult behavioral responses over 2–4 days) before a weather event registers as a change in human incidence.
3. Short-term lags of 3 and 7 days carry the strongest predictive signal. The 7-day lag for wind speed and precipitation aligns with the lower bound of the extrinsic incubation period and is the most biologically defensible. The 3-day surface pressure signal is better interpreted as a habitat trigger acting on an already-primed vector population. Neither lag implies weather-to-case causation within days; both reflect the compounded biology of vector breeding, and viral incubation.
4. Among target-derived features, rolling standard deviation (8.36%) outperforms rolling mean (6.86%). Variance in case counts rises before the mean rises. This is the early-warning signature of epidemic transition described in the dynamical systems literature [61,62]. A monitoring system that watches for increasing case-count variability will detect the pre-outbreak signal that mean-based surveillance misses.
5. Calendar indicators (month, quarter, day of week) contribute 8.57%, a distant third behind meteorological and target-derived features. Seasonal timing matters, but only as a weak prior. The specific meteorological conditions within a season drive transmission; the calendar position alone does not.
6. The feature importance distribution is right-skewed. Most variables cluster below 2% accuracy contribution, while a handful of predictors account for the bulk of the signal. In practice, a parsimonious model built around the top 10–15 features would likely retain most of the predictive accuracy of the full 86-feature set, a practically important result for resource-limited surveillance systems.
7. Surface pressure at a 3-day lag (7.15%) is the single most informative predictor, outpacing the second-ranked feature by more than 4 percentage points. Its primacy is unexpected given how rarely surface pressure appears in the dengue forecasting literature, and it warrants dedicated attention: low-pressure systems create the microclimatic conditions, reduced wind shear, elevated humidity, thermal stability under which *Ae. aegypti* adults are most active and transmission is most efficient.
8. Wind speed and minimum temperature rank as the strongest non-lagged meteorological predictors. Wind speed directly constrains *Ae. aegypti* flight and host-seeking behavior; minimum temperature sets the lower bound on larval development rate and adult survival through cool nights. Both are well-supported by field entomology [26,57,58].
9. Long-lag climate signals (>90 days) contribute minimally, with the sole exception of maximum temperature at lag 180. This exception likely reflects cumulative thermal effects on vector population density across the dry season. Otherwise, the data are consistent: dengue forecasting in Bangladesh operates on short ecological timescales, and early warning systems should be built around near-real-time weather monitoring rather than seasonal climate projections.

Dengue forecasting in Bangladesh can be viewed as influenced by climate, affected by time delays, and responsive to short-term changes and seasonal patterns. A small group of key weather indicators offers the most accurate signals for predicting outbreaks.

## Discussion

Dengue has become a major public health issue in Bangladesh, especially highlighted by the unprecedented epidemic of 2023 [2,26,27,31]. This situation shows the urgent need for effective forecasting tools. Traditional models often rely on combined monthly case counts and limited weather data. They also depend on set assumptions, which do not capture the complex nature of dengue transmission [26]. To tackle this problem, this study uses a detailed method that combines high-resolution daily epidemiological data from the Directorate General of Health Services with thirteen weather variables from NASA and the Bangladesh Agricultural Research Council. The SBD algorithm downscale the data. It divided the total case counts into daily numbers. Extensive feature engineering introduced lagged weather predictors, transformations derived from the target, and time indicators. These features were normalized and organized into sequences with a sliding-window method. Various deep learning models were used, including ANN, LSTM, GRU, BiLSTM, BiGRU, and CNN-RNN hybrid ensembles. The study optimized these models with Bayesian hyperparameter tuning. Next, we used the SSFS algorithm to choose the most important predictors. This entire process aimed to improve forecasting accuracy and to identify the ecological and temporal factors that are key for predicting dengue transmission in Bangladesh. The ANN’s performance advantage over recurrent and attention-based models warrants a methodological note that goes beyond the Results section. A recurring interpretation risk in comparative deep learning studies is that a performance gap in a single dataset is generalized into a claim about model families. We have been careful not to make that claim, but Fig 8 is stark enough that the temptation deserves direct resistance. The gap between ANN (97.05% accuracy) and LSTM (74.54%) is large, but it is best understood as a measurement of how much the feature engineering pipeline has already done. The SSFS-selected feature set encodes 3-day and 7-day lagged meteorological signals, rolling variance terms, and seasonal structure precisely the temporal patterns that LSTM gating is designed to extract from unprocessed sequences. Feeding these pre-computed features into an LSTM is, in a sense, asking a specialist to redo work that has already been done; the feedforward network, which has no architectural prior about sequence order, processes all features simultaneously and fits the regression surface more efficiently under these conditions. This does not generalize to settings with raw data, short observation periods, or sparse meteorological covariates, where recurrent models typically outperform feedforward alternatives. Dataset characteristics (resolution, feature engineering depth, sequence length, and variable count) should drive architecture selection, not cross-study performance rankings.

Bangladesh recorded 321,179 dengue cases and 1,705 deaths in 2023, its worst outbreak on record [2,31]. The central question this study addresses is not which deep learning architecture performs best, but whether short-term meteorological signals, routinely available can provide actionable advance warning of transmission surges. The answer, based on the ANN model’s 97.05% accuracy on held-out data, is yes and the warning window is measurable. Surface pressure shifts, precipitation, and maximum wind speed at lags of 3–7 days collectively explain the dominant share of predictive accuracy, meaning that vector control teams, fumigation scheduling, and hospital surge preparation could, in principle, be triggered by weather thresholds rather than by waiting for case counts to rise.

The study’s findings enhance our understanding of dengue forecasting in Bangladesh. They show how deep learning models can explain the complex links between weather, time, and disease factors. Unlike much earlier research that mostly relied on statistical methods or basic machine learning, this analysis emphasizes the important role of weather variables, especially their past versions, in affecting transmission patterns.

A key part of this research is the detailed assessment of feature importance in several areas. The results in Figs 11 to 16 reveal a clear ranking: weather variables primarily drive predictive accuracy, lagged features consistently outperform current ones, and short-term lags of three to seven days are the most important predictors. These findings match well with known epidemiological principles. The life cycle of the *Aedes aegypti* mosquito, from larval development to viral incubation, takes place over these specific timeframes [60]. This explains why short-lagged weather variables are closely tied to outbreak risks.

The three leading lagged predictors: surface pressure at lag 3 (7.15%), maximum wind speed at lag 7 (2.14%), and precipitation at lag 7 (2.02%) each have a plausible biological pathway that the raw statistics do not make explicit.

Surface pressure at a 3-day lag is the strongest single feature in the model, and this warrants more than passing acknowledgment. Low surface pressure systems over Bangladesh are associated with reduced boundary-layer turbulence, elevated relative humidity, and suppressed wind shear. Under these microclimatic conditions, *Ae. aegypti* adults experience lower desiccation stress during diurnal resting, extend their effective flight range, and increase the frequency of host-seeking attempts [63]. The 3-day lag positions this effect precisely at the behavioral response window: gravid females that become active under favorable pressure conditions reach peak host contact and therefore peak virus transmission, roughly 48–72 hours after the atmospheric shift. It is worth noting that surface pressure is seldom included in dengue forecast models, partly because its mechanism is less intuitive than temperature or rainfall. That it ranks first here, by a margin of more than 5 percentage points over the next predictor, suggests it has been systematically undervalued in the literature.

Precipitation at lag 7 reflects a well-established mechanism. *Ae. aegypti* breeds almost exclusively in man-made containers: water storage vessels, discarded tyres, plant pots, construction debris that fill during rainfall events. At warm-season temperatures (28–32°C), the period from egg deposition to adult emergence runs 5–8 days [64]. A precipitation event therefore delivers a new cohort of biting adults to the population approximately one week later, consistent with the 7-day lag recovered here. This is one of the cleaner quantitative confirmations in the dataset: the model has, without being told, learned the species’ development time from environmental inputs alone.

Maximum wind speed at lag 7 is more nuanced. High wind events suppress *Ae. aegypti* flight directly [65]. A calm period following a high-wind event would therefore correspond to resumed biting activity and elevated transmission risk, with the 7-day lag capturing both the suppression and the rebound. The presence of maximum rather than mean wind speed as the relevant predictor is consistent with this interpretation: it is the magnitude of the inhibitory event, not the average wind load, that determines how sharply activity is subsequently restored.

Although they are less influential than weather-related variables, target-derived features still added value. Rolling window transformations based on variability measures like standard deviation often outperformed those based on means. This fits the pattern of dengue outbreaks, where changes in cases give more information than average numbers. Interestingly, rolling windows showed a bimodal importance structure: very short periods (two days) and longer seasonal spans (180–450 days) added value, while medium periods offered little. This shows that capturing both small changes and larger seasonal patterns maximizes predictive benefits. In contrast, intermediate timeframes do not provide unique information.

The uneven distribution of feature contributions, as seen in Fig 13, highlights that dengue forecasting relies on a small set of key predictors. While many features improve model strength, only a few contribute most of the predictive power. This finding has key implications for methods. Simple models using these key variables can reach similar accuracy as more complex models. They are also easier to understand and faster to run. These models are particularly helpful in low-resource areas, where technical and infrastructure challenges can hinder the use of more complex systems.

These findings also impact policy. The presence of short-term lagged weather variables indicates the need to include real-time weather data in dengue monitoring systems. For instance, changes in surface pressure, rainfall, and wind speed could act as early warnings. This information can help mobilize vector-control teams, target fumigation efforts, and prepare hospitals quickly. The emphasis on variability rather than averages suggests that public health monitoring should focus on measures of irregularity and changes in both weather and case data, rather than solely on average values. Three concrete surveillance implications follow from these findings. Real-time monitoring of surface pressure and wind speed should be integrated into existing dengue early warning dashboards at the divisional level. Public health alerts should be calibrated to variability in weather and case data rather than averages; standard deviation-based rolling features outperformed mean-based ones, suggesting that irregularity, not magnitude, is the more sensitive trigger. The 3-to-7-day warning window, while short, is operationally meaningful: it is sufficient to mobilize community-level larval source reduction campaigns and pre-position clinical supplies ahead of a detected surge.

The question of usable forecast lead time deserves explicit treatment. As configured, the model generates one-day-ahead predictions from a 14-day input window, a *t* + 1 architecture optimized for accuracy, not operational anticipation. In a strict forecasting sense, the system confirms what is about to happen rather than warning what will happen next week. This is a real constraint, and health authorities considering deployment should understand it.

That said, the feature importance results extend the operationally meaningful anticipation window beyond *t* + 1. Surface pressure at a 3-day lag, precipitation at a 7-day lag, and maximum wind speed at a 7-day lag are the three dominant predictors and all three are directly observable meteorological variables available in near-real time from the Bangladesh Meteorological Department and NASA POWER. A practitioner need not wait for case counts to trigger a response. The meteorological trigger itself is the early signal. Concretely, the lag structure recovered here supports the following approximate alert logic. A sustained drop in surface pressure below climatological norms for the season, persisting for 24–48 hours, indicates elevated adult *Ae. aegypti* activity within approximately 3 days and elevated transmission risk within 4–5 days. Rainfall exceeding the monthly $75^{th}$ percentile threshold on any given day indicates a new adult emergence cohort arriving approximately 7 days later, under prevailing warm-season temperatures in Dhaka. A calm period (maximum wind speed falling below 1.5 m/s over 24 hours) following a high-wind event indicates resumed host-seeking activity within 3–5 days.

None of these thresholds are formally calibrated from this dataset that would require a dedicated decision-threshold analysis beyond the present scope, but they are grounded in the lag structure the model has recovered and in the *Ae. aegypti* biological literature. They should be treated as hypotheses for a prospective validation study rather than deployment-ready criteria.

Practical constraints in Bangladesh temper this framework further. Any operational alert system would need to account for these reporting lags explicitly, a model trained on complete, clean daily data will encounter messier inputs in the field. These infrastructure gaps are not reasons to abandon the framework; they are the specific engineering problems that a translational implementation effort would need to solve first.

These insights also be applied on a global scale. Regions where dengue is common, like Southeast Asia, Latin America, and sub-Saharan Africa, have similar ecological and climate conditions, making these findings relevant in those areas. While local adjustments of lags and thresholds will be necessary, the main idea that dengue is driven by climate, influenced by lags, and sensitive to variability offers a strong foundation for developing forecasting systems in different epidemiological contexts. Global health organizations and regional policy groups could use these insights to create standardized weather-integrated monitoring protocols. This would improve readiness for future outbreaks.

This study shows the importance of working together across different sectors. To forecast and prevent dengue effectively, meteorological services, health departments, local authorities, and community groups must collaborate. By including short-term lagged climate signals in decision-making, public health systems can move from reacting to problems to preventing them. This change is especially important in light of climate change, which is likely to increase the variability and intensity of weather patterns, leading to more frequent and severe dengue outbreaks.

This study shows that the factors influencing dengue cases in Bangladesh form a layered structure. Climate is the main factor. Lags shape its effects, while variability points to unpredictable changes. The key takeaway is that forecasting systems that focus on several important lagged weather variables, along with measurements of case variability, can significantly improve readiness for outbreaks. These insights, while based on research in Bangladesh, have value worldwide and offer a guide for data-driven, climate-aware public health policies in the fight against dengue.

## Conclusion

This study shows that dengue forecasting in Bangladesh can be significantly improved by using high-resolution weather data along with deep learning techniques and careful feature design. The findings indicate that dengue patterns are driven by climate, affected by delays, and sensitive to variations. Short-term lagged variables, especially surface pressure, precipitation, and wind speed, are particularly strong predictors. Features that capture variability improved accuracy, while time-based indicators were less important. The uneven distribution of feature contributions shows that a small number of predictors can reach good accuracy. This allows for simple, understandable models that are practical.

However, the study has its limitations. It did not include factors like urbanization, mobility, and vector control practices. While the method using downscaled case data is solid, it may not completely capture reporting details. Future studies should include social and environmental data, consider multiple countries. Connecting forecasts to support tools could also turn predictions into effective strategies for managing outbreaks. Future work could also examine distributional assumptions governing dengue count data. Given the pronounced zero-inflation during inter-epidemic periods (partially addressed here through a sparse binary indicator), negative binomial or zero-inflated Poisson likelihoods embedded within probabilistic forecasting frameworks may better capture the generative process underlying dengue dynamics in Bangladesh. A natural extension is the application of accumulated local effects analysis to quantify how individual meteorological predictors shape dengue risk across their observed ranges and across seasonal strata. This requires either a reformulated pipeline using gradient-boosted or probabilistic models on the same engineered feature set, or a custom marginal effect estimator built directly against Keras model outputs, both tractable under dedicated conditions that the current GPU-accelerated forecasting framework did not accommodate.

In conclusion, this work links predictive modeling with public health by demonstrating how a specific group of past climate predictors and sensitivity to changes can assist in preparing for dengue. While it focuses on Bangladesh, its findings are relevant globally and provide a flexible way to predict dengue as climate change accelerates.

## Supporting information

S1 File The supporting information file titled ‘S1_File.pdf’ depicts the detailed algorithm for Stochastic Bayesian Downscaling (SBD). (PDF)

S2 File The supporting information file titled ‘S2_File.pdf’ depicts the Granger’s causality test p value of the features for varying lag for the target variable in case of the downscaled data. (PDF)

S3 File The supporting information file titled ‘S3_File.pdf’ depicts the topologies of the DNN models adopted in the study. (PDF)

S4 File The supporting information file titled ‘S4_File.pdf’ depicts the detailed algorithm for Sequential Squeeze Feature Selection (SSFS). (PDF)
