# Supplementary material for: Modelling climatic and temporal dynamics of dengue transmission in Bangladesh using deep learning models
Source: PLOS Glob Public Health. 2026 Jul 13;6(7):e0006405. doi: 10.1371/journal.pgph.0006405 (PMC13362098; doi:10.1371/journal.pgph.0006405)
Supplement: S1 File — (PDF) [file pgph.0006405.s001.pdf]

Supporting Information 1: Stochastic Bayesian Downscaling (SBD) Algorithm

---

Algorithm 1: Stochastic Bayesian Downscaling (SBD) Algorithm

Require: Aggregated value vector, $\mathbf{v}$
Overthrow tolerance, δ
Iteration limit, n
The radius of the open interval, r
Standard deviation, σ
Ensure: downscaled time series, $\bar{v}$
for elem in $\mathbf{v}$ **do**
$\bar{v}$ = Distribution Generator(elem,σ)
end for
for i from 1 to n **do**
find a vector of coordinates of overthrow points
for elem in overthrow points **do**
open interval centering elem of radius, r = Distribution Generator(sum of the elements of open interval,σ)
end for
end for
for elem in $\mathbf{v}$ **do**
if $v_i \neq$sum of equivalent aggregate in $\bar{v}$ **then**
d=v_i-sum of equivalent aggregate in $\bar{v}$
while $d \neq 0$ **do**
if $d > 0$ **then**
$\bar{v}_{randomly\, picked\, index} = \bar{v}_{randomly\, picked\, index} + 1$
$d- = 1$
else
$\bar{v}_{randomly\, picked\, index} = \bar{v}_{randomly\, picked\, index} - 1$
$d- = 1$
end if
end while
end if
end for

Algorithm 2: Distribution Generator

Require: Total sum of the downscaled distribution, s
Standard deviation, σ
Ensure: Down scaled approximation over the length of the aggregate, $\bar{v}$
$\bar{v}$ =Fit the decided distribution to the given downscaled time frame
if elems in $\bar{v} < 0$ **then**
$\bar{v} = \bar{v} + |min(\bar{v})|$
end if
if elems in $\bar{v}$ are not integer **then**
$\bar{v} = round(\bar{v})$
end if
if $s \neq \sum \bar{v}$ **then**
d=$s - \sum \bar{v}$

```
    while d ≠ 0 do
        if d > 0 then
            v̄_{randomly picked index} = v̄_{randomly picked index} + 1
            d− = 1
        else
            v̄_{randomly picked index} = v̄_{randomly picked index} − 1
            d− = 1
        end if
    end while
end if
```
