# Supplementary material for: Modelling climatic and temporal dynamics of dengue transmission in Bangladesh using deep learning models
Source: PLOS Glob Public Health. 2026 Jul 13;6(7):e0006405. doi: 10.1371/journal.pgph.0006405 (PMC13362098; doi:10.1371/journal.pgph.0006405)
Supplement: S2 File — (PDF) [file pgph.0006405.s002.pdf]

Supporting Information 2: Granger Causality Test Results

Table 1: Granger's causality test p value of the features for varying lag for the target variable in case of the downscaled data.

	Features												
**Lag**	**max_temp**	**max_wind_speed**	**min_temp**	**min_wind_speed**	**precipitation**	**range_wind_speed**	**relative_humidity**	**specific_humidity**	**sunshine (hour)**	**surface_pressure**	**temp**	**wind_direction**	**wind_speed**
1	0.253173	0.247046	0.031465	0.331101	0.130275	0.336084	0.060446	0.014762	0.00515	0.045472	0.067619	0.585149	0.327937
2	0.236614	0.085565	0.082253	0.08102	0.009657	0.340459	0.127595	0.035353	0.022489	0.058909	0.116484	0.667267	0.07337
3	0.072076	0.086953	0.10124	0.007689	0.076807	0.261947	0.268925	0.054925	0.03383	0.012735	0.063596	0.889002	0.020864
7	0.001466	2.72E-08	0.000896	1.24E-06	7.69E-05	9.33E-07	0.361683	0.000357	0.00194	1.35E-08	0.000276	0.770402	1.32E-07
30	0.451931	0.051857	0.978906	0.315571	6.71E-08	0.131409	0.995586	0.978306	0.328514	0.44502	0.597496	0.98289	0.073674
120	0.118904	0.001331	0.999786	0.026608	8.44E-10	0.054187	0.951087	0.896701	0.658729	0.166973	0.597315	0.991677	4.61E-05
180	0.002107	0.011468	0.993479	0.203546	5.29E-05	0.153703	0.050395	0.488973	0.578281	0.089082	0.604586	0.999538	0.003311
365	0.054031	0.181547	0.948174	0.596635	0.028834	0.010475	0.230662	0.765844	0.546655	0.092094	0.62919	0.000109	0.145991
548	0.069694	0.019499	0.924238	0.010183	0.013512	0.002024	0.195632	0.854495	0.685786	0.254736	0.635181	0.004383	0.006346

The table depicts the p value of the Granger's causality test for varying lagged features for the target variable, Infected(i.e. the number of DENV cases reported each day). For level of significance, $\alpha = 0.05$ the green highlighted cells are the relevant lag for which we reject the null hypothesis, H_0.
