# Supplementary material for: Modelling climatic and temporal dynamics of dengue transmission in Bangladesh using deep learning models
Source: PLOS Glob Public Health. 2026 Jul 13;6(7):e0006405. doi: 10.1371/journal.pgph.0006405 (PMC13362098; doi:10.1371/journal.pgph.0006405)
Supplement: S3 File — (PDF) [file pgph.0006405.s003.pdf]

Supporting Information 3: Deep Learning Model Architectures

S1 Topology of ANN Model

Table 1: Topology of ANN model.

Layer	Output	Activation	Dropout	Learning Rate
Dense	(1, 160)	elu	0.056	$6.99E^{-03}$
Dense	(1, 192)	elu	0.104	
Dense	(1, 192)	elu	0.098	
Dense	(1, 128)	-	-	
Dense	(1, 1)	-	-	

S2 Topology of the LSTM Model

Table 2: Topology of LSTM model.

Layer	Output	Activation	Dropout	Learning Rate
LSTM	(1, 14, 256)	relu	0.25	$1.00E^{-05}$
LSTM	(1, 14, 128)	relu	0	
LSTM	(1, 14, 192)	relu	0.25	
LSTM	(1, 32)	elu	0	
Dense	(1,1)	-	-	

S3 Topology of the GRU Model

Table 3: Topology of GRU model.

Layer	Output	Activation	Dropout	Learning Rate
GRU	(1, 14, 160)	tanh	0.02	$1.48E^{-04}$
GRU	(1, 14, 224)	elu	0.06	
GRU	(1, 14, 128)	tanh	0.14	
GRU	(1, 256)	tanh	0.16	
Dense	(1,1)	-	-	

S4 Topology of the BiLSTM Model

Table 4: Topology of BiLSTM model.

Layer	Output	Activation	Dropout	Learning Rate
LSTM	(1, 14, 224)	tanh	0.14	$5.21E^{-03}$
BiLSTM	(1, 14, 256)	elu	0.19	
BiLSTM	(1, 14, 160)	tanh	0.13	
BiLSTM	(1, 14, 128)	tanh	0.07	
Dense	(1, 1)	-	-	

S5 Topology of the BiGRU Model

Table 5: Topology of BiGRU model.

Layer	Output	Activation	Dropout	Learning Rate
GRU	(1, 14, 224)	elu	0.07	$3.35E^{-04}$
BiGRU	(1, 14, 384)	tanh	0.15	
BiGRU	(1, 14, 320)	tanh	0.21	
BiGRU	(1, 128)	elu	0.05	
Dense	(1,1)	-	-	

S6 Topology of the LSTM-ATT Model

Table 6: Topology of LSTM-ATT model.

Layer	Output	Activation	Dropout	Learning Rate
LSTM	(1, 14, 64)	relu	0.06	
LSTM	(1, 14, 128)	tanh	0.04	
LSTM	(1, 14, 128)	tanh	0.18	$8.70E^{-03}$
ATT	(1, 14, 128)	-	-	
LSTM	(1, 14, 192)	tanh	0.24	
Dense	(1, 1)	-	-	

S7 Topology of the GRU-ATT Model

Table 7: Topology of GRU-ATT model.

Layer	Output	Activation	Dropout	Learning Rate
GRU	(1, 14, 224)	elu	0.07	
GRU	(1, 14, 192)	tanh	0.15	
GRU	(1, 14, 160)	tanh	0.21	
ATT	(1, 14, 160)	-	-	$33.5E^{-04}$
GRU	(1, 256)	elu	0.05	
Dense	(1,1)	-	-	

S8 Topology of the CNN_LSTM

Table 8: Topology of CNN_LSTM model.

Layer	Output	Activation	Dropout	Learning Rate
Convulation	(1, 13, 128)	relu	-	
Polling	(1, 4, 128)	-	-	
LSTM	(1, 4, 96)	elu	0.11	5.07E-04
LSTM	(1, 4, 96)	tanh	0.14	
LSTM	(1, 64)	relu	0.04	
Dense	(1,1)	-	-	

S9 Topology of the CNN_GRU Model

Table 9: Topology of CNN_GRU model.

Layer	Output	Activation	Dropout	Learning Rate
Convulation	(1, 12, 96)	relu	-	
Polling	(1, 6, 96)	-	-	
GRU	(1, 6, 96)	relu	0.22	5.40E-03
GRU	(1, 6, 256)	tanh	0.09	
GRU	(1, 32)	elu	0.07	
Dense	(1,1)	-	-	

S10 Topology of the CNN_BiLSTM Model

Table 10: Topology of CNN_BiLSTM model.

Layer	Output	Activation	Dropout	Learning Rate
Convulation	(1, 12, 32)	elu	-	
Polling	(1, 3, 32)	-	-	
LSTM	(1, 3, 96)	elu	0.08	3.74E-05
BiLSTM	(1, 3, 384)	elu	0.21	
BiLSTM	(1, 320)	tanh	0.17	
Dense	(1,1)	-	-	
