# Supplementary material for: Modelling climatic and temporal dynamics of dengue transmission in Bangladesh using deep learning models
Source: PLOS Glob Public Health. 2026 Jul 13;6(7):e0006405. doi: 10.1371/journal.pgph.0006405 (PMC13362098; doi:10.1371/journal.pgph.0006405)
Supplement: S4 File — (PDF) [file pgph.0006405.s004.pdf]

Supporting Information 4: Sequential Squeeze Feature Selection (SSFS) Algorithm

Algorithm 1 Sequential Squeeze Feature Selection (SSFS)

Require: Dataset F, Model Pipeline $model\_p$, Threshold ϵ

Initialize $E_k \leftarrow [\,]$
Initialize $fi \leftarrow [\,]$
while $min(fi) < \epsilon$ **do**
    while $min(fi\_backward) < \epsilon$ **do**
        Initialize $fi\_backward \leftarrow [\,]$
        $F_k = F - E_{k-1}$
        $A_{F_k} = model_p(F_k)$
        for $f_i \in F_k$ **do**
            $A_{F_k - \{f_i\}} = model_p(F_k - \{f_i\})$
            $fi\_backward$.append$(A_{F_k} - A_{F_k - \{f_i\}})$
        end for
        Find f_{i^*} such that $z_k(f_{i^*}) = min(fi\_backward)$
        if $z_k(f_{i^*}) < \epsilon$ **then**
            E_k.append(f_{i^*})
        end if
    end while
    Initialize $fi\_forward \leftarrow [\,]$
    $F_k = F - E_{k-1}$
    $A_{F_k} = model_p(F_k)$
    for $f_i \in E_k$ **do**
        $A_{F_k \cup \{f_i\}} = model_p(F_k \cup \{f_i\})$
        if $A_{F_k \cup \{f_i\}} - A_{F_k} > \epsilon$ **then**
            $fi\_forward$.append$(A_{F_k \cup \{f_i\}} - A_{F_k})$
            $F_k.append(f_i)$
            E_k.drop(f_i)
        end if
    end for
    $fi \leftarrow$ concatenate$(fi_backward, fi_forward)$
end while
$F^* = F - E^*$
